# Treatment of Adolescent and Young Adults with Acute Lymphoblastic Leukemia

**DOI:** 10.4084/MJHID.2014.052

**Published:** 2014-07-02

**Authors:** Josep-Maria Ribera, Jordi Ribera, Eulàlia Genescà

**Affiliations:** 1 Clinical Hematology Department. Institut Català d’Oncologia-Hospital Universitari Germans Trias i Pujol Universitat Autònoma de Barcelona. PETHEMA Group, Spain; 2 Jose Carreras Leukemia Research Institute. Badalona, Spain

## Abstract

The primary objective of this review was to update and discuss the current concepts and the results of the treatment of acute lymphoblastic leukemia (ALL) in adolescents and young adults (AYA). After a brief consideration of the epidemiologic and clinicobiologic characteristics of ALL in the AYA population, the main retrospective comparative studies stating the superiority of pediatric over adult-based protocols were reviewed. The most important prospective studies in young adults using pediatric inspired or pediatric unmodified protocols were also reviewed emphasizing their feasibility at least up to the age of 40 yr and their promising results, with event-free survival rates of 60–65% or greater. Results of trials from pediatric groups have shown that the unfavourable prognosis of adolescents is no more adequate. The majority of the older adolescents with ALL can be cured with risk-adjusted and minimal residual disease-guided intensive chemotherapy, without stem cell transplantation. However, some specific subgroups, which are more frequent in adolescents than in children (e.g., early pre-T, iAMP21, and BCR-ABL-like), deserve particular attention. In summary, the advances in treatment of ALL in adolescents have been translated to young adults, and that explains the significant improvement in survival of these patients in recent years.

## Introduction

Acute lymphoblastic leukemia (ALL) encompasses a heterogeneous group of disorders. The results of clinical trials in adults have been disappointing compared with those of the pediatric age group, with cure rates of 90% in children compared with 30%–40% in adults^1^. An analysis of the Surveillance, Epidemiology, and End Results (SEER) database showed an improvement in survival in adults over the last two decades, with the greatest significant improvement being in the adolescent and young adult (AYA) group.^2^,^3^

ALL is relatively rare among the AYA populations, whereas it is the most commonly diagnosed leukemia in childhood, accounting for 75% of leukemias diagnosed in pediatric patients. Many retrospective analyses of the adolescent age group with newly diagnosed ALL treated according to either pediatric or adult protocols showed a statistically significantly superior outcome for patients treated with pediatric regimens. A lower relapse rate accounted for the superior results observed in adolescents treated according to pediatric protocols. For that reason, treatment of ALL in AYA has gained increasing interest in recent years. This review will focus on the biology and treatment of ALL in AYA.

## The Concept of AYA

Although there is not a uniformly accepted definition of age subgroups in ALL, it can be considered that classic pediatric ALL patients range from 0 to 14 yr of age, adolescents from 15 to 19 years, young adults from 20 to 39 years, adults from 40 to 60 years and older adults and elderly patients include those beyond the age of 65 years. Thus, AYA encompasses those patients from 15 to 39 years of age.

## Biological Factors in ALL of AYA

Several clinical and biologic characteristics of ALL used for risk stratification and prognostication (e.g., phenotype, cytogenetics and molecular genetics) are age-dependent (Table 1). Regarding the immunophenotype, T-cell ALL (T-ALL) is more frequent in AYA than in children and is known to be associated with slightly poor outcomes. In addition, the “early T-cell precursor ALL” (ETP-ALL) subtype, which is associated with poor treatment response, is frequently presented in AYA.^4^

One of the substantial differences between children and AYA is the difference in cytogenetics and molecular genetics. The genetic abnormalities associated with good prognosis decrease with age. Hyperdiploidy and the t(12;21) [*ETV6-RUNX1*] translocation decrease with older age,^5^ during poor-risk cytogenetics, such as t(9;22) [*BCR-ABL1*], complex karyotype, and hypodiploidy increase in prevalence with age. In addition, several reports have demonstrated that good risk cytogenetics is associated with inferior survival in adults and in adolescents compared with children.^6^ A recent study including a large cohort of teenagers and young adults enrolled in the UKALL2003 and UKALLXII trials showed a higher frequency of IgH@ translocations in AYA.^7^ Although these translocations are associated with an adverse outcome in adults, they are not independent prognostic factors in children and adolescents.

To date, there has been no specific study of genomic profiling of AYA with ALL because these patients have been included in both pediatric and adult ALL studies. However, *JAK* mutations, *CRLF2* alteration, iAMP21 and *BCR-ABL*-like profile are among the frequent alterations reported in AYA, and all of them are associated with a poor prognosis. In summary, the genetic profile of AYA ALL patients seems to be similar to patients with high-risk ALL, suggesting that distinct underlying genetic and biologic characteristics account for part of the inferior results observed in AYA.^8^

In addition to the difference in biological factors between ALL in children and in AYA, there is evidence that adult ALL cells are less susceptible to chemotherapy both *in vitro* and *in vivo*. In some studies ALL patients between 15–30 years of age had a significantly higher minimal residual disease (MRD) burden compared with children.^9^,^10^

As far as host factors are concerned, several features are present in less young patients, being responsible for increased treatment toxicity. They include differences in the metabolism of chemotherapeutic agents, depleted marrow reserve and increased extramedullary toxicity. All these issues increase the frequency of life-threatening infections, organ failure, treatment delays and dose reductions in planned chemotherapy.

## Pediatric-based vs. Adult-based Treatments. Retrospective Studies

Several retrospective reports have shown that adolescents (15–20 yr.) and young adults treated by adult oncologists or hematologists with adult ALL protocols have poorer outcomes than similarly aged patients treated by pediatricians with pediatric protocols despite having the same biologic disease.^11^–^20^ The cut-off point of age for treatment of patients in pediatric or adult hemato-oncology units varies among different countries but usually ranges between 15 and 18 years.

The first study, in which such different outcomes were reported, was conducted in France.^12^ A comparison of AYA aged 15–20 yr, treated with the pediatric-based protocol FRALLE-93 (n=77) with patients of the same age and comparable clinical and biologic characteristics of ALL, who received the adult-based protocol LALA-94 (n=100), showed a complete remission (CR) rate of 94% vs. 83%, respectively. After a median follow-up of 3.5 yr, the supposed event-free survival (EFS) was 67% vs. 41% at 5 years. Multivariate analysis showed an independent influence of the protocol on the outcome. The differences in the drugs employed and, especially in the dose-intensity, might explain the better results of the FRALLE-93 protocol. In this protocol the cumulated dose of prednisone was five-fold higher, the vinca alkaloids three-fold and the asparaginase 20 fold-higher than in the LALA-94 study. In addition, in the FRALLE-93 study the dose of prednisone in induction was higher, and asparaginase was also given in this period, in contrast with the LALA-94 trial. Moreover, the time interval between CR and post-remission therapy was 2 days in the FRALLE-93 vs. 7 days in the LALA-94 study.

The North-American Cancer and Acute Leukemia Group B (CALGB) and the Children’s Cancer Group (CCG) performed a retrospective comparison of presenting features, planned treatment, CR rate, and outcome of 321 AYA, aged 16 to 20 years, which we treated in consecutive trials in either the CCG or the CALGB from 1988 to 2001.^11^ CR rates were identical, being 90% for both the CALGB and CCG AYA. The CCG AYA had a 63% EFS and 67% overall survival (OS) at 7 years in contrast to the CALGB AYA, in which the 7-year EFS was only 34%, and the OS was 46%. While the CALGB AYA aged 16 to 17 years achieved similar outcomes to all the CCG AYA with a 7-year EFS of 55%, the EFS for 18- to 20-year-old CALGB patients was only 29%. Comparison of the regimens showed that the CCG AYA received more intensive central nervous system prophylaxis and higher cumulative doses of non myelosuppressive agents earlier. There were no differences in outcomes in those who reached maintenance therapy on time compared with those who were delayed.

A similar Dutch study in patients aged 15–21 yr yielded similar results,^13^ with a 5-yr EFS of 69% for comparable patients treated with the most dose-intensive pediatric protocol DCOG vs. 34% for those treated with adult protocols ALL-5 and ALL-18 from the HOVON Group. Likewise, comparative retrospective studies from Italy also showed a poorer prognosis for patients aged 14–18 yr treated with adult-type protocols.^14^ In turn, a Swedish study compared patients aged 10–40 yr treated with the pediatric trial NOPHO-92 (n=144) vs. a similar group of patients included in the Swedish Adult ALL Group (n=99).^15^ Significantly higher CR rate (99% vs. 90%) and EFS were observed in patients treated with the pediatric protocol, with the type of treatment being an independent prognostic variable on multivariate analysis. However, it is of note that adults aged 26–40 yr had significantly poorer prognosis than AYA (15–25 yr). Another study from Denmark yielded similar results.^16^ In a retrospective study from the British Medical Research Council (MRC), performed only in adolescents (15–17 yr), who were enrolled in the ALL97/revised99 (pediatric, n=61) or UKALLXII/E2993 (adult, n = 67) trials between 1997 and 2002 (17), the EFS (65% vs. 49%) was higher, and the rate of death in remission was lower in patients enrolled in the pediatric trial.^17^ In a retrospective study conducted in the Princess Margaret Hospital of Toronto restricted to AYA with T-ALL 40 patients (median age 30 yr, range 17–69) were treated with several adult type protocols and were compared with 32 patients (median age 32 yr, range 17–64) treated with a DFCI protocol.^18^ Although there were no differences in CR attainment (93% vs. 84%), the OS and relapse-free survival (RFS) probabilities were significantly higher in patients treated with the DFCI trial (83% vs. .56% and 88% vs. 23%, respectively). On multivariate analysis the treatment group (DFCI vs. non-DFCI) was the major prognostic factor influencing both RFS and OS. Other studies from different countries 19 have shown similar results (Table 2).

Only one population-based study from Finland showed that the outcomes of AYA with ALL treated with pediatric or adult protocols were equal.^20^ One hundred and twenty-eight patients (10–16 yr.) were treated with the pediatric Nordic (NOPHO) protocols and 97 patients (17–25 yr.) with Finnish Leukemia Group National protocols. All patients were centrally referred and treated in five academic centers. The 5-year EFS was 67% for the pediatric treatment group and 60% for the adult treatment group. There were no significant differences in the cumulative doses of corticosteroids, vincristine and asparaginase between the pediatric and adult protocols, whereas the pediatric protocols used a higher cumulative dose of methotrexate and lower doses of anthracyclines than the adult protocols. Epipodophyllotoxins and mitoxantrone were not included in the pediatric protocols. The authors attributed the similar results to the similarity of the pediatric and adult protocols and to the centralized care of the patients in five academic centers, ensuring good compliance and adherence to the protocols in both the two distinct age groups.

Finally, the retrospective data from the MD Anderson Cancer Center using the Hyper-CVAD regimen (not including asparaginase) have also reported favorable results in 102 AYA (median age 19 yr), with CR of 97% and OS of 65%. Preliminary reports from 60 AYA patients aged 12–40 yr treated at the MD Anderson Cancer Center with modified augmented Berlin-Frankfurt-Münster (BFM) therapy showed very promising results (2-yr DFS and OS probabilities of 85% and 91%, respectively) in the subset of patients younger than 25 yr,^21^ stressing the importance of treating these patients in large referral centers.

In summary, the 5- to 6-yr EFS rate for AYA treated with pediatric regimens ranges from 65% to 70% vs. 35% to 50% for adult regimens in almost all retrospective comparative studies. However, it is of note that the former included adolescents with a median age 16 or 17 yr. and the latter were adolescents and young adults with a median age 19 yr. or more. On the other hand, all the studies mentioned above have focused on patients aged 15–21 years, but few have evaluated the results in young adults up to 30 years or more, in which the frequency of patients with adverse prognostic factors is progressively increasing.

A meta-analysis of trials was conducted comparing AYA patients treated with pediatric versus adult regimens.^22^ This meta-analysis included a total of 11 such trials and 2489 patients. The AYA patients treated with pediatric regimens had significantly lower all-cause mortality at three years (relative risk [RR] 0.58, 0.51–0.67). The CR rate was significantly higher when AYA patients received the pediatric regimen (RR 1.05, 1.01–1.1), and there was a significant improvement in the 3-year EFS (RR 1.66, 1.39–1.99). The relapse rate was also lower in patients receiving pediatric-inspired regimens (RR 0.51, 0.39–0.66) with similar non-relapse mortality (RR 0.53, 0.19–1.48).

Several factors may have contributed to explain this different outcome, which cannot be exclusively interpreted in view of some existing differences in the distribution of prognostic factors between the populations enrolled in pediatric and adult trials. A major role is certainly played by differences in protocol design and treatment intensity, with pediatric protocols including more non myelosuppressive drugs with demonstrated activity on ALL blasts, such as asparaginase, glucocorticoids and vincristine. Moreover, central nervous system prophylaxis was administered earlier, with greater frequency and for a more prolonged period in pediatric trials and the duration of maintenance therapy is shorter in some adult trials.^23^–^25^ A more accurate administration of therapy in pediatric Institutions may also play a role, due to a peculiar attitude of pediatricians concerning the need to maintain the doses and schedules prescribed, and a possibly better compliance of adolescent patients treated in a pediatric facility.

## Results of Treatment of Adolescents with ALL by Pediatric Groups

The outcome of adolescents with ALL worldwide has significantly improved over time. Barry et al. reported the outcome of adolescents treated in the Dana-Farber Cancer Institute (DFCI) ALL Consortium Protocols conducted from 1991 and 2000.^26^ A total of 844 patients aged 1 to 18 years, with newly diagnosed ALL were enrolled into two consecutive DFCI-ALL Consortium Protocols. Outcomes were compared in three age groups: children aged 1 to 10 years (n = 685), young adolescents aged 10 to 15 years (n = 108), and older adolescents aged 15 to 18 years (n = 51). With a median follow-up of 6.5 years, the 5-year EFS for those aged 1 to 10 years was 85%, compared with 77% for those aged 10 to 15 years, and 78% for those aged 15 to 18 years. There was no difference in the rate of treatment-related complications between the 10- to 15-year and 15- to 18-year age groups. The 5-year EFS of 78% is superior to published outcomes for similarly aged patients treated with other pediatric and adult ALL regimens.

Nachman et al. reported the results of the CCG1961 trial including AYA up to 21 yr.^27^ The EFS and overall survival (OS) rates were 71.5% and 77.5%, respectively. Rapid responder patients randomly assigned to augmented therapy had a 5-year EFS of 81.8% vs 66.8% for patients receiving standard therapy, but 1 versus 2 interim maintenance and delayed intensification courses had no significant impact on EFS. A WBC count over 50×109/L was an adverse prognostic factor. Given the excellent outcomes achieved with this chemotherapy there seems to be no role for the routine use of stem cell transplantation (SCT) in first remission.

In turn, the results of the total therapy studies XIIIA, XIIIB, XIV 1 and XV from St Jude Children’s Research Hospital in the US including 963 pediatric patients, 89 of whom were older adolescents (aged 15 to 18 yr.), have been published.^28^ In the first three studies the 44 older adolescents had a significantly poorer EFS and OS than the 403 younger patients. On the contrary, in study XV (that included the level of MRD to guide treatment, with featured intensive methotrexate, vincristine, glucocorticoid and asparaginase and early triple intrathecal chemotherapy for higher risk ALL) the EFS of 45 older adolescents was 86.4%, similar to 87.4% for the 453 younger children. The OS was also comparable (87.9% vs. 94.1%, respectively). The authors concluded that older adolescents with ALL can be cured with risk-adjusted intensive chemotherapy without SCT.

Data from the Nordic NOPHO ALL92 protocol showed an EFS lower for patients >10 yr with B-precursor ALL and WBC<50×10^9^/L (71% vs. 83%). Interestingly, for adolescents remaining in remission the mean WBC count during maintenance therapy was correlated with the risk of relapse, being this risk more pronounced in adolescents than in non-adolescents.^29^ Thus, compliance to maintenance therapy may influence the risk of relapse in adolescents with ALL.

In summary, with modern approaches of treatment of ALL with pediatric-based protocols prognosis of adolescents cannot be considered unfavorable since, hopefully, the improvement of pediatric patients can be translated to young adults. This concept has led some groups^30^ to perform a treatment reduction, on the basis of rapid clearance of MRD by the end of induction therapy, also in young adults (up to 25 yr) with standard-risk ALL.

## Pediatric-inspired and Unmodified Pediatric Treatments in Young Adults. Prospective Studies

Several prospective clinical trials using pediatric regimens for adults have been published (Table 3) or are currently ongoing. These studies are divided into two types according to their regimens and patients: a pediatric-inspired protocol planned by dose reduction of a pediatric protocol for adults up to 50 or 60 yr, and an unmodified pediatric protocol for AYA up to 30–40 years.

### Pediatric-inspired protocols

The French GRAALL group reported the results of the pediatric-inspired GRAALL-2003 study including 215 patients aged 15–60 years.^31^ In this study there was an 8.6-fold, 3.7-fold and 16-fold increase in cumulative doses of prednisone, vincristine and asparaginase, respectively, compared with the previous adult-based LALA-94 protocol, although the GRAALL-2003 trial retained some adult options, such as allogeneic SCT for patients with high-risk ALL. The CR rate was 93.5%, and at 42 months the EFS and OS rates were 55% and 60%, respectively. The CR rate, EFS and OS compared favorably with the previous LALA-94 experience. It is of note, however, that in patients over 45 yr there was a higher cumulative incidence of chemotherapy-related deaths (23% vs. 5%) and deaths in first CR (22% vs. 5%), although the incidence of relapse remained stable (30 vs. 32%). The results of this study suggest that the pediatric-inspired therapy is feasible in young adults with ALL in whom the outcome clearly improves at least until the age of 45 yr.

The HOVON Group published the results of a study in adults with ALL up to the age of 40 yr.^32^, inspired by a pediatric regimen (FRALLE approach for high-risk ALL), including intensified treatment with allogeneic SCT. Allogeneic SCT was offered to the standard-risk patients with sibling donor and to all high-risk patients. Fifty-four patients were included, with a median age of 26 yr. Complete remission was achieved in 49 patients (91%), of whom 33 (61%) completed treatment as scheduled. Side effects primarily consisted of infections and occurred in 40% of patients. With a median follow-up of 32 months, the estimated EFS was 66% and OS 72% at 24 months. In turn, the Princess Margaret Hospital used a modified Dana-Farber Cancer Institute pediatric protocol in 68 adult patients (17 to 71 years), with a CR rate of 85% and 3-year OS and DFS of 65% and 77%, respectively.^33^

### Unmodified pediatric protocols

The Spanish PETHEMA study was the first trial to compare the results of the unmodified pediatric protocol ALL96 in adolescents (15–18yr., n=35) and young adults (18–30 yr., n=46) with standard-risk (SR) ALL.^34^ Both groups were comparable for the main clinical and biologic characteristics of ALL. The CR rate was 98% and after a median follow-up of 4.2 yr., 6-year EFS and OS were 61% and 69%, with no differences between adolescents and young adults. No significant differences were observed in the timing of treatment delivery, although the hematologic toxicity in consolidation and reinforcement cycles was higher in young adults than in adolescents. These results suggest that unmodified pediatric protocols can be efficiently and safely employed in adult patients with SR ALL, at least up to the age of 30 yr. Similarly, an Australian study has recently reported the results of the FRALLE-93 pediatric protocol in 40 AYA up to 45 years, with no treatment-related mortality and an OS probability of 75% in patients with SR ALL, without the need for allogeneic SCT.^35^

However, until recently, the efficacy of a fully pediatric protocol for AYA with high-risk ALL has not been analyzed. The Japan Adult Leukemia Study Group (JALSG) conducted a phase 2 trial in which 129 patients aged 15 to 24 with *BCR-ABL*-negative ALL were treated with the same protocol developed for children with ALL by the Japan Association of Childhood Leukemia Study (JACLS). The CR rate was 94% and the 5-year DFS and OS rates were 67% (95% CI 58–75%) and 73% (95% CI 64–80%), respectively.^36^ Severe adverse events frequently occurred but the frequency was similar to that observed in children treated with the same protocol. Only insufficient maintenance therapy significantly worsened the DFS.

The DFCI Combined Adult/Pediatric ALL Consortium has applied a pediatric protocol for adults aged 18–50 years.^37^ Specifically, the investigators used an extended course of asparaginase for 30 weeks. The results in 74 patients have shown a CR rate of 82% with promising 2-year EFS and OS probabilities of 72.5% and 73.2%, respectively. This study proved that extended asparaginase treatment was feasible in adults, and the drug-related toxicity was manageable, although the incidence of pancreatitis (13%) and thrombosis/embolism (19%) was a matter of concern. The University of South California group^38^ used an augmented BFM pediatric regimen with eight doses of pegylated asparaginase to treat adults with ALL aged 19–57 years (median 33), with a 3-year projected EFS of 65%. Toxicity attributable to asparaginase was frequent but manageable. However, older patients showed significantly less tolerance to asparaginase, vincristine and steroids compared to children or adolescents. In the FRALLE group from France 30 Philadelphia chromosome-negative adult ALL patients 16 to 57 years of age were treated in the FRALLE 2000 protocol consisting of a prednisone pre-phase and a 4-drug induction including asparaginase, consolidation, delayed intensification and maintenance chemotherapy. The 4-yr DFS was 90% vs. 47% in matched historical controls.^39^

The undergoing U.S. intergroup trial C10403 (available at www.clinicaltrials.gov. NCT00558519) has been developed to examine and describe results among AYA with newly diagnosed ALL, treated using a successful Children’s Oncology Group regimen (COG ALL0232), which give rise to 78% survival rate for older adolescents aged 16–21 years. Individuals aged 16–39 with newly diagnosed ALL are eligible for participation.^40^ This trial will test the feasibility of treating young adults up to the age of 40 with a pediatric regimen, assess adherence by patients and adult oncologists, and describe the toxicities observed. A component of this trial will analyze issues based on demographics, psychosocial characteristics, and pretreatment features of the disease that are unique to the AYA population. The results of this trial will be compared with patients up to 29 years of age who were enrolled and treated by pediatric oncologists on the COG AALL0232 study (now closed to accrual). The goal of this trial is to demonstrate that the adult cancer cooperative groups can deliver a “true pediatric” regimen to AYA patients and achieve similar outcomes.

In recent years, the MRD research has been incorporated to the therapeutic trials of adult AL, and it has been shown that a subgroup of patients with good MRD clearance can be successfully treated without SCT.^41^–^43^ Although these trials incorporate elements of pediatric protocols, no specific data on the AYA subpopulation were presented to date.

## Allogeneic Stem Cell Transplantation (SCT) in AYA

The role of allogeneic SCT in AYA is not clearly defined, and there are no prospective trials to assess the role of allogeneic SCT specifically in this population. An older retrospective study, from 1995, included patients between the ages of 15–45, from the International Bone Marrow Transplant Registry, and showed no survival advantages compared with chemotherapy alone. Transplanted patients had lower relapse rates, but this was offset by the higher transplant-related mortality.^44^

The largest published study evaluating the role of allogeneic SCT in ALL was a joint effort of Medical Research Council (MRC) in Great Britain and Eastern Cooperative Oncology Group (ECOG).^45^ This trial enrolled nearly 2000 ALL patients and 234 patients younger than 20 years of age. Based on this trial, there was an improvement in the 5-year OS of all patients (53% vs. 45%) and standard-risk patients with Philadelphia-negative ALL (62% vs. 52%). The 10-year cumulative relapse rate was 24% when allogeneic SCT was utilized versus 49% when patients were treated with chemotherapy alone or autologous SCT. However, there was no significant survival advantage in the high-risk group (41% vs. 35%) (*P* = 0.2). The high transplant-related mortality in this group (36%) offset the lower relapse rate in the high-risk group. One of the major limitations of this study was the use of adult regimens in treating AYA. A meta-analysis from 13 studies including 2962 patients, excluding Philadelphia chromosome-positive patients, showed a survival benefit for having a matched sibling donor for patients < 35 years of age (OR = 0.79; 95% CI, 0.70–0.90, P = .0003) but not for those ≥ 35 years of age (OR = 1.01; 95% CI, 0.85–1.19, P = .9) and concluded that matched sibling donor myeloablative SCT improves survival only for younger patients, with an absolute benefit of approximately 10% at 5 years.^46^ Improved chemotherapy outcomes and reduced nonrelapse mortality associated with allogeneic SCT may change the relative effects of these treatments in the future. A recently published retrospective study compared changes in survival after myeloablative SCT for ALL among children (n = 981), AYA (n = 1218), and older adults (n = 469) who underwent transplantation over 3 time periods: 1990 to 1995, 1996 to 2001, and 2002 to 2007. Survival improved over time in AYA and paralleled that seen in children and was primarily related to lower rates of early treatment-related mortality in the most recent era, whereas relapse rates did not change over time.^47^ A comparative study of outcomes of children and AYA undergoing allo-HCT for B-ALL showed that AYA had a significantly inferior survival and a greater transplantation-related mortality compared with children aged <13 years, but with no differences in relapse, suggesting that allo-SCT may overcome relapse in AYA.^48^

Therefore, the use of allogeneic SCT in standard-risk AYA patients remains controversial and warrants further investigation.^49^ As MRD analysis plays a central role in defining the indications for allo SCT using pediatric-type programs in adult patients^50^, future studies should focus on more precisely identifying poor-risk features, such as disease genomics and host pharmacogenomics, refining MRD measurements, improving unrelated donor matching, reducing MRD prior to alloHSCT, and developing post-alloHSCT humoral and cellular therapy approaches.^51^

## Figures and Tables

**Table 1:** Biological features in adolescents and young adults (AYA) with acute lymphoblastic leukemia (ALL)

Characteristic	Comment
**Cytogenetics**	Low incidence of: hyperdiploidy, t(12;21) High incidence of: hypodiploidy, t(9;22), other poor risk cytogenetics, IgH@ translocations, complex karyotype
**Molecular genetics**	Increased frequency of mutations of: CRLF2, JAK, IKZF1, CDNK2A/B High incidence of: IAMP21, BCR-ABL-like
**Immunophenotype**	Higher incidence of early T precursor ALL
**Response to chemotherapy**	Higher MRD burden after induction chemotherapy
**Tolerabilty to intensive chemotherapy**	Poorer in some studies

**Table 2:** Retrospective comparative studies in adolescents and young adults with acute lymphoblastic leukemia treated with pediatric (P) vs. adult-based (A) protocols.

Country (reference)	Protocol	Age	N	CR (%)	EFS (%)
USA (11)	CCG(P)	16–20	197	9	63
	CALGB(A)		124	90	34
France (12)	FRALLE93(P)	15–20	77	94	67
	LALA94 (A)		100	83	41
Holland (13)	DCOG (P)	15–18	47	98	69
	HOVON (A)		44	91	34
Italy (14)	AIEOP (P)	14–18	150	94	80
	GIMEMA (A)		95	89	71
Sweden (15)	NOPHO-92(P)	10–40	144	99	65
	Adult (A)		99	90	48
UK (17)	ALL97 (P)	15–17	61	98	65
	UKALLXII(A)		67	94	49
Mexico (19)	LALIN (P)	15–25	20	90	70
	LALA (A)		20	80	40
Finland (20)	NOPHO (P)	10–25	128	96	67
	ALL (A)		97	97	60

N: number of patients; CR: complete remission. EFS: event-free survival

**Table 3:** Prospective studies in adolescents and young adults with acute lymphoblastic leukemia treated with pediatric-based or-inspired protocols.

Country (reference)	Protocol	Age	N	CR (%)	EFS (%)
USA (36)	DFCI 91-01, 95-01	15–18*	51*	94	78
Spain (34)	PETHEMA ALL-96	15–18	35	94	60
		19–30	46	100	63
France (31)	GRAALL-2003	15–45	172	95	58
USA (37)	DFCI	18–50	74	82	72.5**
Canada (33)	Modified DFCI	17–71	68	85	65***
France (38)	FRALLE-2000	16–57	30	90	90****
Holland-Belgium(32)	HOVON 70	17–39	54	91	66**

* Results restricted to adolescents;

** Estimated at 2 years;

*** Overall survival.

**** Disease-free survival.
